# Report upon the Rise, Progress and Prospects of Mechanical Dentistry

**Published:** 1854-07

**Authors:** M. D. French


### ARTICLE III.

*Report upon the Rise, Progress and Prospects of Mechanical Dentistry, read before the Associated Alumni of American Dental Colleges*, at the Baltimore College, March 18, 1854. By M. D. FRENCH, D. D. S.

AT a meeting held in this place in March, 1853, for the purpose of organizing a dental society, to be called the Associated Alumni of American Dental Colleges, you did me the honor to appoint me to prepare and read, a t your second annual meeting, a report on mechanical dentistry. I am fully sensible of the important and responsible nature of the duty, which this flattering expression of confidence has imposed, and the consciousness of my inexperience and inability to do the subject justice, tends to augment the natural embarrassment, and occasion much anxiety that my efforts should meet your approval; if, however, an earnestness of purpose and an honest desire to contribute to the advancement of this department of science, can give these efforts any merit, I shall at least have some slight claim upon your favor.

Before entering upon a review of the various appliances necessary for the correction of irregularities in the arrangement of the natural teeth, of the different methods adopted in the construction of artificial substitutes, of palates, obturators, &c., and the manipulations connected therewith, I shall make some general remarks relating to the history and progress of this important branch of our profession.

It must be a source of no small gratification to every lover of the profession to know, that in the last thirty or forty years, a period fruitful in improvements and discoveries, which the philosophers of any former period would have deemed incredible, dental science has taken the lead in the onward march, and achieved triumphs of which the annals of any other profession scarcely afford an example.

Previous to this time the history of dentistry is but the record of ignorance and quackery. Despised and neglected by men of science, it fell into the hands of barbers and mountebanks, whose knavery and presumption was only equalled by their ignorance, and thus a noble art, capable in its present state of excellence, of conferring inestimable blessings upon mankind, became, in the hands of such practitioners, a formidable instrument of disease and torture. Totally destitute of all the essential qualifications of the dentist, and having no other claim upon the confidence and patronage of the public, or right to perform operations requiring manual dexterity, combined with a knowledge of anatomy and pathology, than that which they might urge by virtue of their having assumed the title, they pursued a system of ignorant and barbarous practices which the ancient Egyptian, could he have come forth from his catacomb and witnessed, might have blushed to own was characteristic of the time of his own existence.

Yet in the midst of this chaos of ignorance, dentistry engaged the attention of a few individuals whose talent and genius shown out as brilliant meteors amid the surrounding intellectual darkness, and whose works gave to the profession a certain degree of importance; still, comparatively, little was done to develop its hidden resources and raise it from the degraded position in which it had so long slumbered, until a period within the memory of men still in the profession.

Most of the theoretical dental knowledge then possessed was derived from the works of the celebrated John Hunter. His treatise upon the teeth, opened up a wide field for subsequent study and investigation, and the novel and interesting nature of the subject naturally attracted the scientific and curious.

Such men as Fox, Delabarre and others, whom I might mention, devoted their energies to the advancement of the science.

Yet dental surgery cannot be said to have taken the position to which its importance so justly entitled it, until the time of Mr. Bell, who wrote in 1829.

His work upon the anatomy, physiology and pathology of the teeth, by demonstrating them to form an integral part of the animal economy, and pointing out the important relations and sympathies which they maintain with the surrounding and distant parts, thus showing a knowledge of them to constitute an essential element of a thorough medical education, gave to the study and practice of dentistry an importance which it had never before attained.

Men, justly famed for their talent and learning, medical men skilled in their profession, turned their attention to it, and devoted their energies to the augmentation of dental science, and under the patronage of such men it rapidly advanced in public confidence and respectability. Although a majority of the profession has ever been, and still is, composed of persons whose pretensions to science and professional worth are but base mockeries, the number of her educated members has rapidly increased, and dentistry has gradually won its way to a rank among the liberal professions.

It is a lamentable fact, that for many years, while dental anatomy, pathology and surgery were rapidly advancing in importance and usefulness, that branch of the profession which relates to the supplying artificial dentures and the correction of irregularities in the arrangement of the natural teeth, seemed to lie in abeyance. Until a very recent period, the knowledge in this department of dentistry was exceedingly limited and imperfect, and it is a matter of surprise and regret that the early writers on the subject should have given so little attention to that branch of the art so absolutely essential to the successful practice of the profession as mechanical dentistry.

Of the French and English writers, Delabarre, Koecker and a few others, were the only authors who have given anything like an accurate description of the principles upon which artificial teeth are inserted, and the various methods adopted in the process of their construction, until Mr. Robinson, a late writer, whose work on mechanical dentistry contains much valuable information.

In 1846, Dr. Solyman Brown wrote a series of papers on this branch of the art, which were published in the American Journal of Dental Science, in which he described very minutely, not only the principles upon which artificial teeth are applied, but all the different methods used in the process of their insertion.

The work of Dr. Brown contained all the information possessed on the subject at the time, and constituted the best and most complete work which had then been published.

Since the publication of Dr. Brown's treatise, there have been numerous important improvements in this branch of the profession, which may be found accurately described by Dr. Harris, in his Principles and Practice of Dental Surgery, a work for its lucid and scientific exposition of dentistry, every way worthy of the experience and high attainments of its author, and which, I take for granted, is in the hands of every man ambitious of excelling in the profession.

Having made these general remarks in order to show the rapid advance which the science has made with a view to a better appreciation of the advantages and facilities enjoyed by the profession at the present time over that of any former period, I shall endeavor to point out, as far as pertains to the mechanical department of the art, what those advantages and facilities are. I shall avoid all minute descriptions except such as may be necessary to indicate the different methods adopted for the attainment of the same end, and to show the advantages of the one over the other.

*The manner of correcting Irregularities of the Teeth.*—The most aggravated and complicated cases of irregularities, if taken while the patient is quite young, may be remedied by a judicious application of mechanical pressure. The principle upon which they are corrected is the same in all instances, and a practical knowledge of that will enable any one possessing skill and ingenuity to adopt his appliance in such a manner as to overcome the most difficult and obstinate irregularities. The proper manner of applying it must be determined by the peculiarities of the case.

It frequently occurs when the irregularity is slight and occasioned by a crowded condition of the teeth, that the removal of the cause will be sufficient to remedy the evil. Where the crowded condition of the teeth renders the removal of any of them necessary, one should be extracted from each side, and when the proper teeth to be removed are not indicated by caries, and the space between the lateral incisor and first bicuspid is equal to one-half the width of the cuspidati, the second bicuspid should be selected; but when the space between the lateral incisor and first bicuspid is less than one-half the width of the cuspidati, the first bicuspid should be removed. Of the various plans proposed for the correction of irregularities in the teeth, the inclined plane of Catalan, and the use of ligatures, by means of a gold bar bent to correspond with the dental circle, as recommended by Mr. Fox, are almost the only means which have come into general use, and are acknowledged to be the best ever adopted.

There is another method which I am not aware has ever been practiced, and which, from experience, I can recommend, confident that it will be found to possess some important advantages, both for comfort and utility, over any other means. This consists of a plate of silver, accurately adapted to the roof of the mouth, and secured with clasps to the molar teeth, and by means of gold springs, or spiral springs, soldered to the plate, a constant and steady pressure may be applied to the teeth in any direction. The arrangement of the springs will, of course, be determined by the peculiar circumstances. In cases where it is necessary to exert a constant outward pressure, spiral springs enclosed in tubes should be soldered to the plate in such a manner that the ends of the springs shall press upon the teeth in the required direction. Now, the advantages of this method over any other are, that it does not require to be rearranged every three or four days, as is the case when ligatures are used; it can be worn with but slight inconvenience, except that occasioned by the pressure on the teeth; it can be taken out and replaced at the will of the person wearing it, and does not occasion that unsightly appearance presented by the gold bar and ligatures.

*Substances used for Artificial Teeth.*—These are human teeth, teeth of cattle and sheep, teeth carved from the ivory of the tusks of the elephant and hippopotamus, and porcelain teeth. Human teeth, and those carved from ivory, have been in use since the earliest history of mechanical dentistry.

For beauty of appearance and congeniality with the mouth, the human teeth, when they are properly chosen and arranged, are, perhaps, superior to any others; they should be selected from the same class as those for which they are to be substituted, and when they are of a dense firm structure, with perfectly sound enamel, they will sometimes last for fifteen or twenty years.

Their susceptibility to the chemical actions of the fluids of the mouth is much greater than that of those having a vital connection with the adjacent parts, and unless they are of good quality, and the mouth in which they are inserted be in a healthy condition, they seldom last longer than from three to five years. Teeth carved from ivory, while they possess none of the advantages ascribed to the human teeth, their white opaque appearance always giving to the countenance a sickly and disgusting aspect, are much more permeable, and consequently more readily destroyed by the acids of the mouth.

By absorbing the fluids of the mouth, they turn black almost immediately, and when many of them are worn in the same mouth, they impart an exceedingly offensive odor to the air which is returned from the lungs, which of itself should constitute an insuperable objection to their use. So numerous and palpable are the objections to all animal substances for artificial teeth, that the leading members of the profession everywhere have abandoned their use, and in this country porcelain teeth have become almost universally adopted. The advantages which mineral teeth possess over all others are confessedly great. They can be more accurately adapted to the mouth and plate; they are not affected by the deleterious fluids of the mouth, and if proper attention is paid to their cleanliness, they never change color, and by the indefatigable labors of a few men, the art of their manufacture has been brought to such a degree of perfection, that they can be made to resemble so closely the natural organs as frequently to escape detection even by the most practiced observer.

*Manner of Securing Artificial Teeth in the Mouth.*—The different methods adopted for the retention of artificial teeth in the mouth are, the engrafting of artificial crowns on the fangs of the natural teeth, inserting them on plates secured by means of clasps, spiral springs and atmospheric pressure.

The plan of inserting artificial teeth upon natural roots has for a long time been in general use, and is, when circumstances are favorable to its application, the best that can be adopted.

The high estimation in which it has ever been held, and the facility with which the operation may be performed, has occasioned many to forget the conditions absolutely necessary to success, and to perform the operation indiscriminately, and too often under circumstances, the nature of which render success impossible.

In order to derive permanent advantage and benefit, the root upon which the tooth is engrafted must be in a healthy condition, and the practice which some have of inserting teeth upon diseased roots, or fangs having diseased sockets, should be repudiated by every respectable dentist. Unfortunately, the incisors and cuspidati of the upper jaw are all that can be replaced in this manner; all the rest, and these, when the roots are gone, require teeth inserted upon a plate.

In most cases of partial sets inserted upon plates, the use of clasps becomes necessary. It is true, that such plates may be retained by atmospheric pressure, but in cases of less than eight or ten teeth, where the arrangement of the remaining natural teeth is favorable, I regard the use of clasps as far preferable to any other method.

In this way the teeth may be much more firmly secured, thereby rendering them more capable of performing the functions of the natural organs which they replace, and when accurately fitted they need occasion but very slight injury to the natural teeth. The use of spiral springs and atmospheric pressure are, except when the suction method is resorted to for the retention of partial upper sets, adapted to cases precisely similar. When they are not used in connection, either one or the other is made to subserve the purpose of the two. When the gums are soft and spongy, or when from disease, they are continually undergoing change in form, spiral springs may be used with advantage, but I regard them as useless and unnecessary in every instance of sound healthy gums. If the plate has a properly constructed air chamber and is accurately adapted to all the inequalities of the gums and palate, success in the operation is inevitable, and if we urge the necessity of springs in such cases we do but acknowledge our inability to fit the plate to the mouth accurately.

*Inserting Pivot Teeth.*—Artificial crowns are secured to the roots of the natural teeth by means of pivots made of wood or metal. When the former is used it should be of the best quality of seasoned hickory, and when the latter, platinum or fine gold should be selected, as any other metal would become more or less oxydized by the fluids of the mouth. Much diversity of opinion exists in regard to the material that should be used for pivots. Some prefer the simple gold pivot, or a metallic pivot encased in a thin layer of wood, and others again, wood to metal in any form.

The use of the metal, and the plan of inserting a hollow gold screw into the canal in the root for the reception of the pivot, I believe is, unquestionably, the best that can be employed; the tube effectually protects the walls from the action of all corrosive agents, thus preventing the enlargement of the canal by decay, as is always the case when the tube is not used, and although it is a more tedious and expensive operation, the advantages which it confessedly possesses, should recommend it to the favorable notice of every practitioner. The success of the operation is rendered still more sure and complete by filling the root from the upper extremity of the tube to the apex of the fang.

A tooth, thus inserted, may be removed, cleansed and replaced at the will of the patient, and if the operation has been skillfully performed, it will stand twice as long as a tooth inserted in any other way.

*Taking an Impression of the Mouth.*—The best materials for this purpose are white or yellow wax and plaster of paris. The former is always used for taking impressions of the lower jaw, and generally for partial sets in both jaws, some even prefer it for full upper sets, but a great majority of dentists give plaster the preference in such cases. The manner of taking a wax impression is too well understood to require any description. Plaster of paris is sometimes used with much advantage for partial sets, but it is more particularly adapted for full upper sets, in which case it is always preferable to wax, the moist plaster, from its soft and yielding nature, requires little pressure in its application, thus securing a very accurate impression, whereas the force necessary to apply the wax must somewhat displace the soft parts and render the impression less perfect. In procuring a plaster impression of the mouth, a plate struck upon a model made from a wax impression, or a wax-holder with a border of wax so attached as to stop up the open ends and increase the depth of the ordinary rim, so that when the plaster is applied to the mouth it will completely encase the alveolar ridge, is generally used for introducing the plaster into the mouth, but the method which I have recently adopted, and which I think possesses some advantages, is to take an impression of the mouth in wax, then remove all the superfluous wax and cut away the wax from the inner side of the rim so as to admit a body of plaster between the outer surface of the alveolar ridge and the wax, and with this proceed to take the plaster impression.

*Getting up Plaster of Paris Casts, Metallic Models and Counter Models.*—It is a very simple affair to take a plaster cast; a batter of plaster of paris has merely to be poured into the impression and allowed to remain there until set.

For procuring a metallic cast and counter cast, various means are adopted. A composition of mercury, bismuth and tin, which melts at a temperature of less than 200° F., is sometimes used.

This prevents the necessity of making a plaster cast, as the compound, when melted, is poured at once into the impression, but as this makes the cast less perfect than either of the following, I prefer them, particularly the latter. Zinc and tin being combined in the proportion of three of zinc and one of tin for the male cast, and lead alone or tin, which is better for the counter cast, should be used.

The first plan is to make a counter model by dipping the plaster cast into the melted lead, and into this counter model the molten zinc and tin is then poured to form the male cast. The other method is to mould the plaster cast in sand and into this mould to pour the melted zinc and tin, which forms a metal cast, which is then dipped into the molten led or tin, and forms the counter cast. Some difficulty occasionally exists in drawing the cast from the sand by reason of the projection of the alveolar ridge, which causes the sand to draw in removing the cast, thereby rendering the metallic cast somewhat imperfect, but these defects can be easily remedied, by, before the metal cast becomes quite cool and is in a soft state, cutting away the parts with some sharp instrument so as to make it correspond to the original plaster model. The difficulties may be avoided altogether by using Dr. George Hawes' recently invented moulding flask.

*Metals for Plates.*—Gold, palladium and platinum are the only metals which should ever be employed as a basis for artificial teeth, because they only are capable of withstanding the chemical actions of the fluids of the mouth, except for temporary sets, when silver may be used with less expense and will answer the purpose quite as well. Gold is almost always used, except when the teeth are united to the plate by means of a silicious cement, in which case the piece has to be subjected to a high degree of temperature and the use of platinum becomes necessary.

Gold, employed as a basis for artificial teeth, should be at least eighteen carats fine for lower and partial sets, and twenty for full upper sets. For upper plates it should be in thickness from 25 to 27 Stub's gauge, and for lower plates, backings and clasps, from 22 to 24.

*Swaging up Plates.*—Before attempting to swage the plate between the casts, it should be partially fitted to the male cast with a wooden hammer; without this precaution, it is liable to mutilate the cast, and thus occasion an imperfect adaptation of the plate. During the process of swaging, the plate should be annealed six or eight times, and if any portion of the zinc or lead should adhere to the plate, it should be carefully removed before annealing, as the fusion of these metals upon its surface would render the gold brittle and liable to crack. To avoid the danger of such an occurrence, it is well to oil the plate before striking it between the dies. After the plate has been perfectly adapted to the cast, it should be fitted to the mouth previous to attaching the teeth and clasps.

*Fitting the Clasps.*—This is an operation requiring nice manipulation, and upon the manner in which it is performed, depends much of the success of the operation.

The clasps having been accurately fitted to the teeth in the patient's mouth, the plate should be adjusted, and the two united by a cement composed of wax, one or two parts, and resin one, previously warmed, and applied to the plate and each clasp. The whole should then be carefully taken from the mouth and imbedded in plaster, after which the cement should be removed and the clasps soldered to the plate.

The clasps and plate are usually fitted and attached on the plaster cast, but as the adaptation cannot be thus made so perfect as when they are fitted to and united in the mouth, I award the latter method a decided preference. It sometimes happens that the teeth do not stand in a vertical position, and it is impossible to remove the plate and clasps when attached with cement, without changing their relative position. In cases of this sort, the plan recommended by Dr. Fogle, of attaching them by means of a strip of gold, about a half an inch long, bent so as to form a semi-circle, soldered to the plate and clasps, may be found very serviceable. Clasps united to the plate in this manner may be removed without difficulty. There is another method suggested by Dr. Noble, which is valuable in cases of this sort, which is the use of plaster of paris, instead of cement, for uniting the clasps to the plate in the mouth, for a description of which, I shall refer you to Dr. Harris' work.

*The manner of Procuring an Antagonizing Model.*—Getting up a model for antagonizing a partial set, when the remaining teeth in one jaw antagonize with those in the other, is a very simple operation. The clasps having been soldered to the plate, a rim of wax is placed upon it, and the whole is applied to the mouth. The patient then closes the mouth until the teeth in the upper and lower jaws come together, imbedding the teeth in the lower jaw in the wax. The piece is then removed, placed upon the plaster model and covered with a batter of plaster of paris, which, by filling the impressions in the wax, made by the teeth in the lower jaw, shows, when dry, the exact relation which those teeth sustain to the teeth in the upper jaw.

For obtaining an antagonizing model for full upper sets, or double sets, the method of placing a rim of soft wax upon the plate, or if a double set, between the two plates, with a piece of wood corresponding in width to the length of the required artificial teeth, introduced through the wax at a point corresponding with the median line, and having the patient close the mouth upon the wax until the teeth and plate, or the two plates, as the case may be, shall strike upon the wood, is very frequently adopted. But as there is considerable uncertainty in regard to the result of this operation, in consequence of the inability of the patient to close the mouth naturally, the following method may be regarded as preferable.

A rim of wax is placed upon the convex surface of the plate, and cut away until it shall correspond in width to the length of the proposed artificial teeth, and also strike the natural teeth in the other jaw on both sides, at the same instant. It is also trimmed on the outer surface, to give the proper contour to the cheeks and lips. This done, it should be removed, and the plaster applied in the same manner as in cases of partial sets.

In procuring an antagonizing model for a complete double set, the wax upon the upper plate is arranged in the same manner as described above.

After having adjusted a rim of warm wax upon the lower plate, they are both applied to the mouth, the wax on the upper plate being cold and hard, and the mouth closed, imbedding the rim on the upper plate in the wax on the lower plate. The impression thus made in the wax will serve as a guide in cutting it away on its outer and inner surface. It should be trimmed, so that when the mouth is closed and the relative position of the jaws perfectly natural, the two waxes shall exactly correspond in thickness, and meet all the way round. After this is accomplished, the two are firmly secured together by means of pins, and the whole removed from the mouth, and the antagonizing model completed by applying the plaster to the plates in the usual manner. In this way, if care is used in trimming the wax, and in closing the mouth, previous to uniting the waxes, the proper relation of the plates on the antagonizer can be secured with unerring accuracy.

*Mounting Single Porcelain Teeth upon a Metallic Base.*— The usual manner of mounting single plate teeth is to attach them to the plate by means of gold standards secured to the backs of the teeth by platinum pins put in the teeth during the process of their manufacture, and soldered to the plate. Without attempting to detail the various methods of proceedure adopted by different dentists in attaching single teeth to a metallic base, I shall endeavor to describe as briefly as possible that plan which is most generally practiced, and which, for simplicity combined with utility, is certainly preferable to any other. The teeth having been accurately fitted to the plate, and arranged according to the antagonizing model, and held in their proper places by a rim of wax on their inner surfaces, a paste of plaster of paris and fine sand, or a batter of equal parts plaster and asbestos should be applied to the palatine surface of the plate and to the outer surface and coronal extremities of the teeth, to the thickness of from a quarter to a half an inch.

To prevent the displacement of the teeth in case the plaster should crack during the process of soldering, the piece and plaster should be enclosed in a hoop made of a strip of sheet iron about three-fourths of an inch wide. When the plaster and asbestos have become dry, the teeth should be removed, one at a time, and a standard placed upon the back of each, and accurately fitted to the plate. The holes should be countersunk on the back part of the plate, so as to permit a head on each pin to prevent their drawing. They should then be secured to the backs of the teeth by uniting the platinum pins with a pair of punching forceps. In doing this, a thin piece of cork should be interposed between the labial surface of the tooth and the instrument, and with the punch on the end of the pin, the force is applied in the same manner as in punching a plate. In this way, the pins can be nicely headed without danger of fracturing the tooth.

Some dentists secure the backings to the teeth before soldering, by bending the pins in opposite directions. When this is done, the standards are only held to the pins by a union with the solder, and unless an unnecessarily large amount of solder is fused upon the surface of the backing, it is much more liable to break, and the rivets draw, than when they are properly headed.

The practice of riveting the pins with a light hammer is even more objectionable than this, because the concussion which attends the use of the hammer not unfrequently starts the pins in the teeth, and although the injury thus occasioned may not be observable at the time, a few months, or even weeks, of actual service, may suffice to bring it to light by the rivets breaking out, and the teeth coming off.

The pins should not be riveted so closely as to prevent the solder from flowing around them and firmly uniting them to the backings. This effect will be rendered the more certain by taking the precaution to put a little borax around the pins previous to heading them.

The teeth all having been backed and readjusted in the plaster, borax and solder should be applied to the pins, and at the line of union between the standards and plate, and the whole soldered at once. In soldering, it is important that the heat should be applied gradually, and in such a manner as to preserve an equal degree of temperature throughout the piece, as the solder flows better, and the plate is much less liable to spring from unequal expansion under such an application of heat, than when one portion of the plate is suddenly heated to a degree of temperature much higher than the rest. Some diversity of opinion exists in regard to the best means of applying heat for the purpose of soldering. A variety of ingenious contrivances have been invented for this purpose, some of which are often used with advantage. The furnace and blow-pipe, invented by Dr. Somerby, of Louisville, Ky., and the self-acting blow-pipe, by Dr. Parmly, of New York, are, perhaps, the best that has ever been given to the profession.

The ordinary spirit lamp and common mouth blow-pipe are the most frequently employed, and to one who is skilled in its use, it is preferable to any other.

*Securing Single Teeth in a Base of Tin.*—The method of constructing an artificial substitute for the natural teeth in the inferior maxilla, by securing the teeth in a base of tin, was first practiced by Dr. Hudson, of Philadelphia, as early as 1820. I am not aware, however, that he ever adopted it generally in his practice. It never had claimed much attention, indeed but very little was known about it until the publication of a treatise upon this subject, in the American Journal of Dental Science, in 1850, by Dr. Hawes, of New York, although Dr. Royce, of Mewbury, New York, had constructed a denture on this plan in 1836. Dr. Hawes, whose experience in this manner of mounting teeth for under sets has been somewhat extensive, believes it, in all cases of whole or fractional under sets of artificial teeth, to be superior to any other method, and since the publication of his article on the subject, it has occasionally been employed by a few dentists. As it is not generally approved of by the profession, I do not deem it necessary to make any remarks upon its merits or demerits.

*Single Plate Teeth mounted upon a Metallic Base, with Continuous Artificial Gums.*—The principle of uniting single porcelain teeth to each other, and to the plate upon which they are set by means of a fusible silicious cement, which flows in and around the base of the teeth, upon the plate, in such a manner as to form a continuous artificial gum, was originated in France, and although it had been practiced there since about the year 1820, it never attained a degree of perfection sufficient to give it any advantage over the ordinary method of mounting single teeth until 1851, when it was introduced into the profession in the United States, almost simultaneously by Dr. John Allen and Dr. W. M. Hunter, both of Cincinnati, Ohio. The composition employed in this style of work is used on plates of pure platinum, or twenty-two carat gold, alloyed with platina.

The teeth and gums are modeled to the plate, and the whole is then subjected to an intense heat, until fusion of gum takes place, when the teeth become firmly united and attached to the plate. By this method, the cavities between the teeth and between the teeth and plate, which are unavoidable in the old style of mounting single teeth, are completely filled up, leaving no chance for the lodgement of extraneous matter more than must always exist in the most perfectly mounted block teeth.

A piece of work can be made in this way in about one-half the time that is required for putting up a set of block teeth, and in the hands of most dentists would be superior in point of beauty, and if the test of experience shall prove that a denture constructed on this plan possesses the requisite strength and durability, it is certainly superior to any which has ever been practiced by the profession, and must, sooner or later, supersede all others. I am not, however, fully satisfied that such will ever be the case, for during the process of mastication, the plate is liable to spring and the gum crack off, and many dentists who adopted it practiced it only to find their high hopes of its ultimate success disappointed, and have hence abandoned it altogether. To what extent these unfortunate results may be attributed to an improper manner of compounding and working the material, or an imperfect adaptation of the plate to the mouth, it is impossible to say.

This objection to this plan of mounting single porcelain teeth we are told can be easily overcome by replacing the loss with composition, and fusing it to the plate, and fractured edges of the remaining portion of the gum. It is true that this may be done once, or even twice, without material injury to the piece, but if the operation is frequently repeated, the color in the gum is bleached by the action of the heat, and a new gum rendered necessary.

The process of restoring the color of the gum by fusing upon its surface a second coat of enamel, is always attended with more or less injury to the denture. The gums frequently assume a dark purple color, and this combined with the fulness around the necks of the teeth, occasioned by the second application of enamel, often gives to them the appearance, when in the mouth, of turgid and swollen gums. Occasionally it happens, that during the process of fusing the enamel, the gum is blown to such an intent, as if not to render them absolutely unfit for use, to give them a most unnatural appearance.

Now, if the fractures which occur in this sort of gum, cannot be repaired except at such imminent risk of injury, or totally destroying the beauty and natural appearance of the whole piece, it would seem that the argument advanced that the facility with which such fracture can be repaired, removes the objection which their occurrence offers to the use of this style of work, loses much of its force, and the repairing process itself is comparatively worthless.

The durability and success of this kind of work, evidently depends upon the strength originally given to the gums, rather than upon the facility with which it can be repaired when broken, and unless science and experience shall give to work put up in this manner a greater amount of strength than has ever yet been obtained, the utility of the method is altogether problematical.

*Block Teeth.*—In no branch of our profession has the march of improvement been more rapid than in the manufacture and insertion of block teeth, and the fact of their general use by the best dentists sufficiently attests the high estimation in which they are almost universally held, and fully justifies the statement that they can be put up in a manner, which for beauty and utility is superior to any other style, the merits of which has been tested by experience. They are adapted to all cases where teeth with gums are required.

They must be manufactured to suit the particular case in which they are to be used, hence it is important that every dentist should make them for his own use, and if he is not able to carve and mount them himself, he should at least have sufficient knowledge of the art to enable him to superintend and direct others in the operation.

The limits of this article render it impossible for me to give any adequate description of the process gone through in the manufacture of these teeth. I shall, therefore, refrain from any attempt to do so, and proceed to notice the different methods of attaching them to the plates.

That most frequently adopted, and which is by a majority of dentists believed.to be the best, consists in securing the blocks to the plate by the use of standards attached to the palatine surface of the blocks, by means of platinum pins put in the blocks before they are biscuited, and soldered to the plate.

The backings may be disconnected the same as when single teeth are used, or form one continuous band on the inner surface of the blocks. A continuous band gives the nicest finish, and it affords less opportunity for the lodgment of extraneous matter than the separate standards, it is probably the better of the two. The beauty and durability of the piece will be greatly enhanced by putting a *rim of gold around their outer surface.*

Another plan which is sometimes employed, is to solder pins on the plate, to correspond to the vertical holes made through the teeth, and the blocks fastened by riveting the pins on the grinding surface of the molars and bicuspids, and on the labial surface of the cuspidati and incisors.

The last method I have to notice is, that of attaching the blocks to the metallic base, by setting them either in gutta percha, or what is better, a cement composed of equal parts of sulphur and fine feldspar upon pins, soldered to the plate, to correspond to the vertical holes in the blocks. The blocks should be accurately fitted to the plate and to each other, and after the pins have been soldered on a strip of gold, about one-half the thickness of the plate and one-eighth of an inch in width, should be fitted and soldered to the plate, so as to form a continuous band around the base of the blocks. Notwithstanding the discredit into which this plan has fallen, I have no hesitation in expressing the belief, that in all cases where the length of the teeth will admit of the use of pins one-eighth of an inch in length, it is the very best that can be employed.

I am aware, that in this, I am giving expression to an opinion conflicting with that entertained by a large majority of dentists, but the results of my own experience have satisfied me, that the objection urged against teeth mounted in this way, that they do not possess sufficient strength and durability, is entirely groundless. That such has been the experience of many is true, but in a vast majority of cases, the fault was in making, and not in the principle.

Many, and indeed, almost every one who has ever adopted this method, have overlooked the importanee of the band around the base of the blocks, either as a support to them, or as a protection to the gutta percha from the action of the fluids of the mouth, and have inserted the teeth without it, and because, in the course of a few months, or even weeks, they had the mortification to see the adhesive property destroyed by chemical action and the teeth come off, a result that scarcely needed experience to teach would be the inevitable consequence of such a practice, they at once abandoned it as inefficient and worthless, whereas, if the band had been used, and the blocks set in a mineral cement, a degree of strength would have been given them quite equal to those mounted with pins and backings. When this method is employed, any springing that may occur in the plate during the process of soldering on the pins and band, can be easily remedied before the teeth are set upon it, by binding the plate with fine wire on the plaster model, and heating it to a red heat while in this position, and thus preserving the original adaptation of the plate to the mouth, but if in soldering on the blocks, the plate should undergo any change in conformation, and its liability to do so is much greater than in the other case, by reason of the unequal expansion by heat of the porcelain and metal, it is exceedingly difficult to restore it to its original shape, and it not unfrequently happens, that the best directed efforts fail to accomplish it, and a partial or total loss of suction is the consequence. Another important advantage which teeth mounted in this manner has, is the facility with which the loss of one or two of the blocks can be replaced without injury to the remaining block or blocks, as the case may be. In order to this, however, it is necessary that the model on which the original teeth were carved should be preserved.

If the blocks have been soldered on, it is with the utmost difficulty that the backings can be removed without springing the plate, and if that is accomplished, the chances are ten to one that the remaining blocks, especially if they have been long worn in the mouth, will fracture during the process of soldering on the new ones, and thus ruin the whole piece.

*Artificial Palates and Obturators.*—Mechanical appliances for remedying the defects in the palatine organs, have for a long time been employed, and although they never can completely restore the functions of the natural parts, they may, if properly applied, in many cases be made to subserve so perfectly the purposes of the natural parts as to render the inconvenience attending their loss comparatively trifling.

When the defect to be repaired consists of a simple opening in the hard palate, the plan adopted for its correction is the one recommended by Bourdet, with the improvement by Delabarre of securing the instrument to the teeth by means of clasps. This consists of a gold plate accurately adapted to the vault of the palate sufficiently large to cover the opening with two lateral arms, one on each side, extending outward to the teeth, to which they are clasped. When the lesion involves the soft palate, and the action of the muscles draw the soft parts away from the plate to prevent the passage of fluids up between the plate and palate into the nasal cavity in deglutition, and also to prevent the accumulation of the mucous secretions of the nose in the cavity above the plate, Delabarre recommends that a metallic drum be soldered on the palatine surface of the plate in such a manner as to nicely fit and completely fill up the opening in the palate. This is a very important improvement, and is absolutely essential to the complete success of the operation in all cases where the velum is implicated, and it may always be employed with decided advantage even when the aperture is confined to the hard palate.

The manner of constructing a plate of this description is very simple. The plate is fitted to the mouth and the clasps to the teeth in a manner as previously described. This done, a wax impression of the hole in the palate is taken, and a plaster of paris cast made, after which a metallic model and counter model is procured, and a gold plate is swaged between the two. After the drum has been accurately adapted to the aperture, it is adjusted on the palatine surface of the plate and soldered to it. It is important that the plate and drum should be nicely fitted to the parts, otherwise it would prove a source of constant irritation, and thus greatly augment the evil which it was designed to remedy.

It occasionally happens that in addition to the plate and drum, art is invoked to supply the loss of a part or the whole of the velum and uvula. For this purpose, a variety of ingenious contrivances have been invented, but, unfortunately, though much permanent advantages has in many instances been derived from the use of artificial substitutes for these parts, none has ever yet been constructed which could perform, to any very considerable extent, their proper functions, or that could be worn without more or less inconvenience to the patient.

The different instruments which have been constructed for supplying these defects are too complicated to admit of a description of them here. They are, however, minutely described by Professor Harris, in his Principles and Practice of Dental Surgery.

